# Rho Associated Coiled-Coil Kinase-1 Regulates Collagen-Induced Phosphatidylserine Exposure in Platelets

**DOI:** 10.1371/journal.pone.0084649

**Published:** 2013-12-16

**Authors:** Swapan K. Dasgupta, Anhquyen Le, Sandra B. Haudek, Mark L. Entman, Rolando E. Rumbaut, Perumal Thiagarajan

**Affiliations:** 1 Center for Translational Research on Inflammatory Diseases, Michael E. DeBakey Veterans Affairs Medical Center, Baylor College of Medicine, Houston, Texas, United States of America; 2 Department of Pathology, Baylor College of Medicine, Houston, Texas, United States of America; 3 Department of Medicine, Baylor College of Medicine, Houston, Texas, United States of America; 4 Department of Pediatrics, Baylor College of Medicine, Houston, Texas, United States of America; University of Leuven, Belgium

## Abstract

**Background:**

The transbilayer movement of phosphatidylserine mediates the platelet procoagulant activity during collagen stimulation. The Rho-associated coiled-coil kinase (ROCK) inhibitor Y-27632 inhibits senescence induced but not activation induced phosphatidylserine exposure. To investigate further the specific mechanisms, we now utilized mice with genetic deletion of the ROCK1 isoform.

**Methods and Results:**

ROCK1-deficient mouse platelets expose significantly more phosphatidylserine and generate more thrombin upon activation with collagen compared to wild-type platelets. There were no significant defects in platelet shape change, aggregation, or calcium response compared to wild-type platelets. Collagen-stimulated ROCK1-deficient platelets also displayed decreased phosphorylation levels of Lim Kinase-1 and cofilin-1. However, there was no reduction in phosphorylation levels of myosin phosphatase subunit-1 (MYPT1) or myosin light chain (MLC). In an *in* *vivo* light/dye-induced endothelial injury/thrombosis model, ROCK1-deficient mice presented a shorter occlusion time in cremasteric venules when compared to wild-type littermates (3.16 ± 1.33 min versus 6.6 ± 2.6 min; p = 0.01).

**Conclusions:**

These studies define ROCK1 as a new regulator for collagen-induced phosphatidylserine exposure in platelets with functional consequences on thrombosis. This effect was downstream of calcium signaling and was mediated by Lim Kinase-1 / cofilin-1-induced cytoskeletal changes.

## Introduction

The Rho-like small GTPases such as RhoA, Rac, and Cdc42 regulate cytoskeletal remodeling by binding to downstream effectors in a variety of cells [1–3]. Two closely related kinases, Rho-associated coiled-coil serine/threonine kinase-1 (ROCK1) and -2 (ROCK2) have been identified as key downstream effectors of RhoA [4]. Though ROCK1 and ROCK2 share 92% amino acid sequence identity across their kinase domains, they have distinct biological effects [5]. In addition, genetic deletion of ROCK2 is embryonically lethal, as ROCK1 cannot compensate for the loss of the other [6]. 

Following vessel wall injury, platelets adhere firmly and rapidly to exposed collagen fibrils in the subendothelial matrix through multiple receptors [7]. These interactions result in transbilayer movement of phosphatidylserine from the inner to the outer leaflet of the membrane bilayer [8,9]. Phosphatidylserine confers a procoagulant surface necessary for hemostasis by providing binding sites for the assembly of prothrombinase and tenase complexes on the surface of activated platelets. Previous studies have shown that the Rho associated coiled-coil kinase (ROCK) inhibitor Y-27632 inhibits senescence induced but not activation induced phosphatidylserine exposure [10]. ROCK signaling has also been associated with platelet shape change [3,11–14]. However, these studies relied on the use of the ATP competitive ROCK kinase inhibitor Y-27632, which does not distinguish between ROCK1 and ROCK2 [15]. Further, Y-27632 has additional off-target inhibitory activity for other kinases [16]. In the current study, we aimed to decipher the specific role of ROCK1 in platelet activation. We used genetically altered mice, deficient in ROCK1 expression, ROCK1^-/-^ mice[17], to explore platelet activation in response to collagen.

We, here, present evidence that in response to collagen stimulation, ROCK1 deficiency caused increased exposure of phosphatidylserine on platelets and concurrent augmented thrombin generation, however, without being involved in shape change, ATP secretion or aggregation. Further, ROCK1-deficient mice have a shorter occlusion time in a light/dye-induced endothelial injury/thrombosis model. These effects were accompanied by diminished phosphorylation levels of Lim Kinase-1 and cofilin-1, and alterations in platelet cytoskeleton. 

## Materials and Methods

### Mice

The generation and maintenance of homozygous ROCK1-deficient (ROCK1^-/-^) mice in an FvB background were described previously [17,18]. ROCK1^-/-^ mice are viable and morphologically indistinguishable from their wild-type littermates. However, the number of ROCK1^-/-^ offspring from heterozygous parent mice was significantly below the normal Mendelian distribution. The investigation involving mice was conformed to the Guide for The Care and Use of Laboratory Animals as published by the US National Institutes of Health. All animals were treated in accordance with the protocol approved by the Animal Care and Use Committee (IACUC) of Baylor College of Medicine.

### Reagents

Collagen (equine tendon collagen) was purchased from Helena Laboratories; thrombin, prothrombin, factor Xa, and factor Va from Hematologic Technologies Inc. Calcium ionophore A23187, apyrase, indomethacin, Y-27632, fluorescein isothiocyanate (FITC)-dextran, and prostaglandin E1 (PEG1) were obtained from Sigma-Aldrich. Latrunculin-A, Alexa Fluor 488-phalloidin and Fura-2 AM were from Invitrogen. Anti-phospho-cofilin-1 (ser 3) and antiphospho-myosin light chain (MLC; threonine 18) antibodies were from Santa Cruz Biotechnology. Anti-phospho-MLC (serine 19), anti-phospho-Lim Kinase-1, and anti-β-actin antibodies were from Cell Signaling Technology. Anti-phospho-myosin phosphatase target subunit-1 (MYPT1) antibody was from Millipore. FITC-lactadherin was generated as described before [19]. Anti-CD42b was purchased from eBioscience. 

### Isolation of Platelets

Blood was obtained from human volunteers after an informed written consent under a protocol approved by the Institutional Review Board of Baylor College of Medicine. Blood was drawn through 19-gauge needles into polypropylene syringes containing 1/10 volume of 3.8% trisodium citrate, pH=6.5, and immediately transferred to polypropylene tubes. Platelets were prepared as previously described [20] and suspended in modified Tyrode’s buffer (137 mM NaCl, 2.7 mM KCl, 5 mM Hepes, 1 mM MgCl2, 3 mM NaH2PO4, 5.5 mM Dextrose, pH=7.4) containing 1% bovine serum albumin. Platelets were stimulated with collagen (10 μg/ml). For inhibition studies, platelets were incubated with Y-27632 (20 μM) or latrunculin A (2 μM) for 20 minutes before stimulation.

To isolate mouse platelets, blood was drawn from the inferior vena cava into 3.8% trisodium citrate from ≥4-month-old mice under the influence of isoflurane. Blood was diluted with an equal volume of HEPES-buffered saline and platelet-rich plasma was obtained by centrifugation at 260 g for 10 minutes. PGE1 (1 µM) was added, and platelets were sedimented by centrifugation at 1000 g for 10 minutes, washed twice in Tyrode’s buffer (pH=6.6), and suspended in Tyrode’s buffer (pH=7.4). 

### Platelet Aggregation

Platelet shape change and aggregation were monitored in a lumi-aggregometer (Chrono-Log) at 37°C and 1200 rpm stirring speed. Secretion of granule ATP was determined simultaneously by adding luciferin-luciferase reagent (Chrono-Lume from Chrono-Log). 

### Platelet Prothrombinase Activity

Prothrombinase activity of collagen-treated platelets was measured as described previously with some modifications [20]. The reaction mixture consisted of prothrombin (1μM), factor Va (10 nM), factor Xa (10 pM), calcium (1.5 mM), collagen (10 μg/ml), and platelets (1x10^6^/ml) in Tyrode’s buffer (pH=7.4). Thrombin formation was determined by measuring absorbance at 405 nm in a microplate reader (BioTek).

### Flow Cytometry

Expression of phosphatidylserine and F-actin were quantified on a flow cytometer (Coulter FCC 500, Beckman-Coulter) using the CXP software as described previously [19]. The gates for intact platelets were set using a fluorescein-conjugated anti-CD42b antibody and light scatter, and fluorescence channels were set at a logarithmic gain. For phosphatidylserine expression, isolated platelets were incubated with FITC-lactadherin (5 µg/ml) and PE-labeled anti-CD42b (2.5 µg/ml) for 30 minutes at room temperature [19]. For F-actin quantification, the resting and activated platelets (3x10^8^/ml) were fixed with 4% paraformaldehyde for 10 minutes and permeabilized with 0.1% Triton X-100 for 10 minutes at room temperature. The platelets were then stained with Alexa Fluor 488-phalloidin as per the manufacturer’s protocol (Invitrogen).

### Intracellular Calcium Studies

Isolated platelets from ROCK1^-/-^ and wild-type mice were incubated with Fura-2 AM (3 µM) for 30 minutes at 37°C, then washed twice with Tyrode’s buffer (pH=6.6) containing PGE1 (1uM), apyrase (10 units/ml), and indomethacin (10uM) and suspended in Tyrode’s buffer (pH=7.4). Fura-2 fluorescence was measured using a Cary Eclipse Fluorescence Spectrophotometer (Varian) with an excitation wavelength alternating from 340 to 380 nm; the emission wavelength was set at 510 nm. Once a stable base line was achieved, collagen (10 μg/ml) and calcium chloride (1 mM) were added and the changes in fluorescence were recorded. In some experiments Fura-2 AM loaded platelets were incubated with Y-27632 (20 μM) or BAPTA-AM (20 μM) for 10 minutes before addition of collagen. The concentration of intracellular calcium was determined from the ratio of Fura-2 fluorescence intensity at 340 and 380 nm by the formula of Gronkiewicz (20). 

### Mouse-Tail bleeding Time

Mouse-tail bleeding time was performed as described [21]. Briefly, mice were placed in a restraining device and using a sharp scalpel, 3 mm of the distal tail was transected. The bleeding time was measured from the moment of transection until the arrest of bleeding.

### Immunoblots

Isolated platelets from ROCK1^−/−^ and wild-type mice were stimulated with collagen (10 µg/ml) and at various time intervals, solubilized in Tris-buffered saline containing 0.25% deoxycholic acid, 1% NP-40, 1 mM EDTA, and a phosphatase / protease inhibitor cocktail mixture (Thermo Scientific). Aliquots (30 - 50 µg of protein) were separated by SDS-PAGE in 8-16% gels (Thermo Scientific), transferred to PVDF membranes, and blotted with primary antibodies against phosphorylated Lim Kinase-1, Cofilin-1, MYPT-1, myosin light chain and β-actin, followed by the appropriate HRP-conjugated secondary antibodies. Band intensities were quantified using Image J software. The densitometric value of each protein was divided by the respective β-actin value. The relative band intensity was calculated as fold-change compared to the unstimulated wild-type sample.

### Mouse Model of Thrombosis

Thrombosis was assessed in a mouse model of light/dye-induced endothelial injury as described previously [22]. Briefly, endothelial cell injury was induced by injecting 10 ml/kg of 5% FITC-conjugated dextran (~150 kDa) and subsequently exposing ~100 μm of the vessel to filtered excitation light at 0.6 W/cm^2^ (from a 175W xenon lamp (Sutter) and an HQ-FITC filter cube (Chroma)) [22]. Epi-illumination was applied continuously. The time of onset of platelet aggregation (thrombus onset) and the time of flow cessation (for at least 60 seconds) were recorded. Thrombi were induced in at least two venules per animal and the results for each animal were averaged. 

### Statistical Analysis

All data are expressed as mean ± standard deviation of triplicate measurements except when indicated otherwise. Comparisons between individual groups were performed using the Student T-test with paired and unpaired samples. A probability value (P) of 0.05 or below was considered statistically significant. 

## Results

### ROCK1 is Not Involved in Collagen-Induced Platelet Shape Change or Aggregation

**Figure 1:**
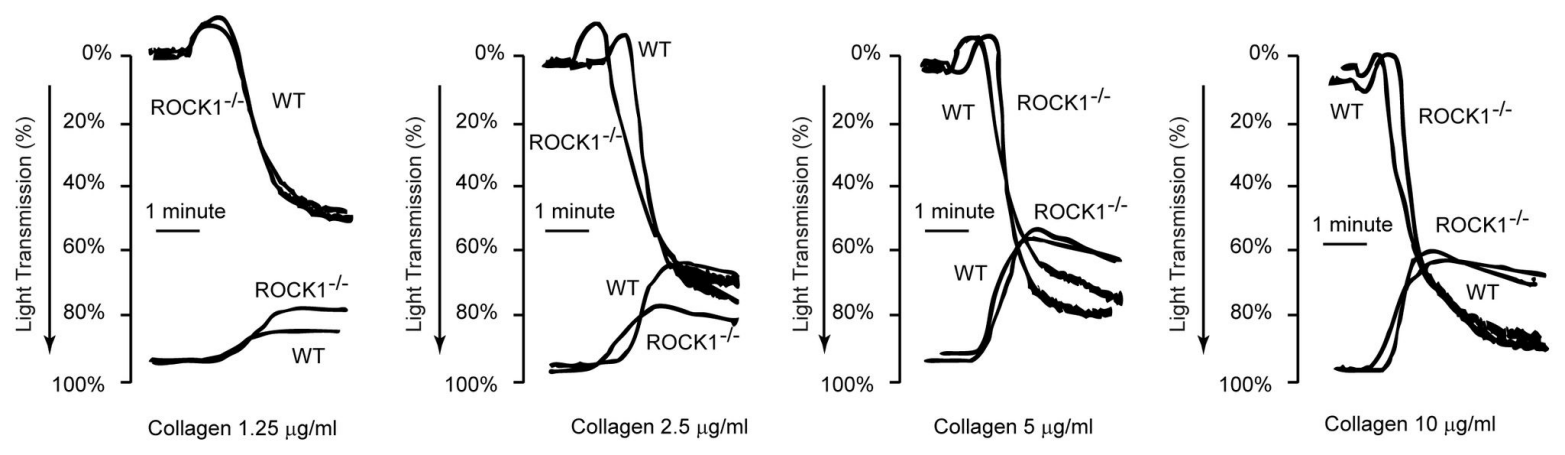
Collagen-induced platelet aggregation and ATP secretion are normal in ROCK1-deficient mice. Platelets were isolated from ROCK1-deficient mice and littermate wild-type controls and stimulated with the indicated concentrations of collagen. Aggregation and ATP secretion were measured in a lumi-aggregometer. The traces shown are representative of at least 3 independent experiments.

**Figure 2:**
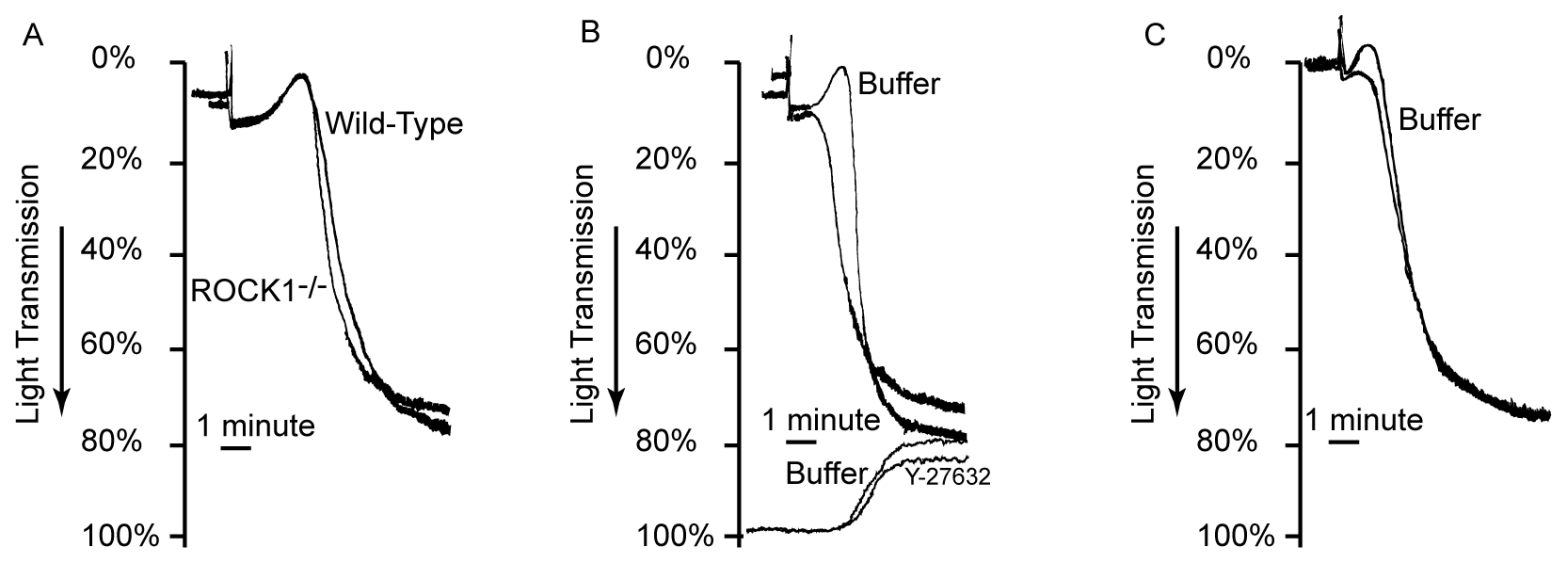
ROCK1 deficiency does not affect platelet shape change. Isolated murine (A, B) or human (C) platelets were stimulated with collagen (10 µg/ml,) and aggregation and ATP release (where indicated) were measured in a lumi aggregometer. (A) Wild-type and ROCK1-deficient platelets responded similarly to collagen. (B) Addition of non-specific ROCK inhibitor Y-27632 to ROCK1-deficient platelets abolished platelet shape change, similar to its effect on human platelets, though it did not alter ATP secretion. (C) The traces shown are representative of at least 3 independent experiments.

The platelet count in ROCK1^-/-^ mice was similar to the count in wild-type littermate controls (1.3 ± 0.3 x10^6^/ml for ROCK1^-/-^ versus 1.2 ± 0.5 x10^6^ ml for wild-type mice). There were no significant differences in the mean platelet volume (5.3 ± 1.0 fl for ROCK1^-/-^ versus 5.2 ± 1.2 fl for wild-type mice). The platelet aggregation and ATP secretion to collagen was similar to wild-type littermate controls (Figure 1). Previous studies have shown that the non-specific ROCK inhibitor Y-27632 inhibits collagen-induced shape changes in human platelets [11,14]. However, in our study using platelets from ROCK1-deficient mice, we found normal shape changes in response to collagen (Figure 2A). However, when we incubated ROCK1-deficient platelets with Y-27632, we found inhibition of shape change similar to those seen in wild-type murine (Figure 2B) and human platelets (Figure 2C). Also, Y-27632 did not alter ATP secretion (Figure 2B). These data indicated that ROCK1 was not involved in collagen-mediated platelet shape change, aggregation, or secretion. 

### ROCK1 is Involved in Phosphatidylserine Exposure and Thrombin Generation

**Figure 3:**
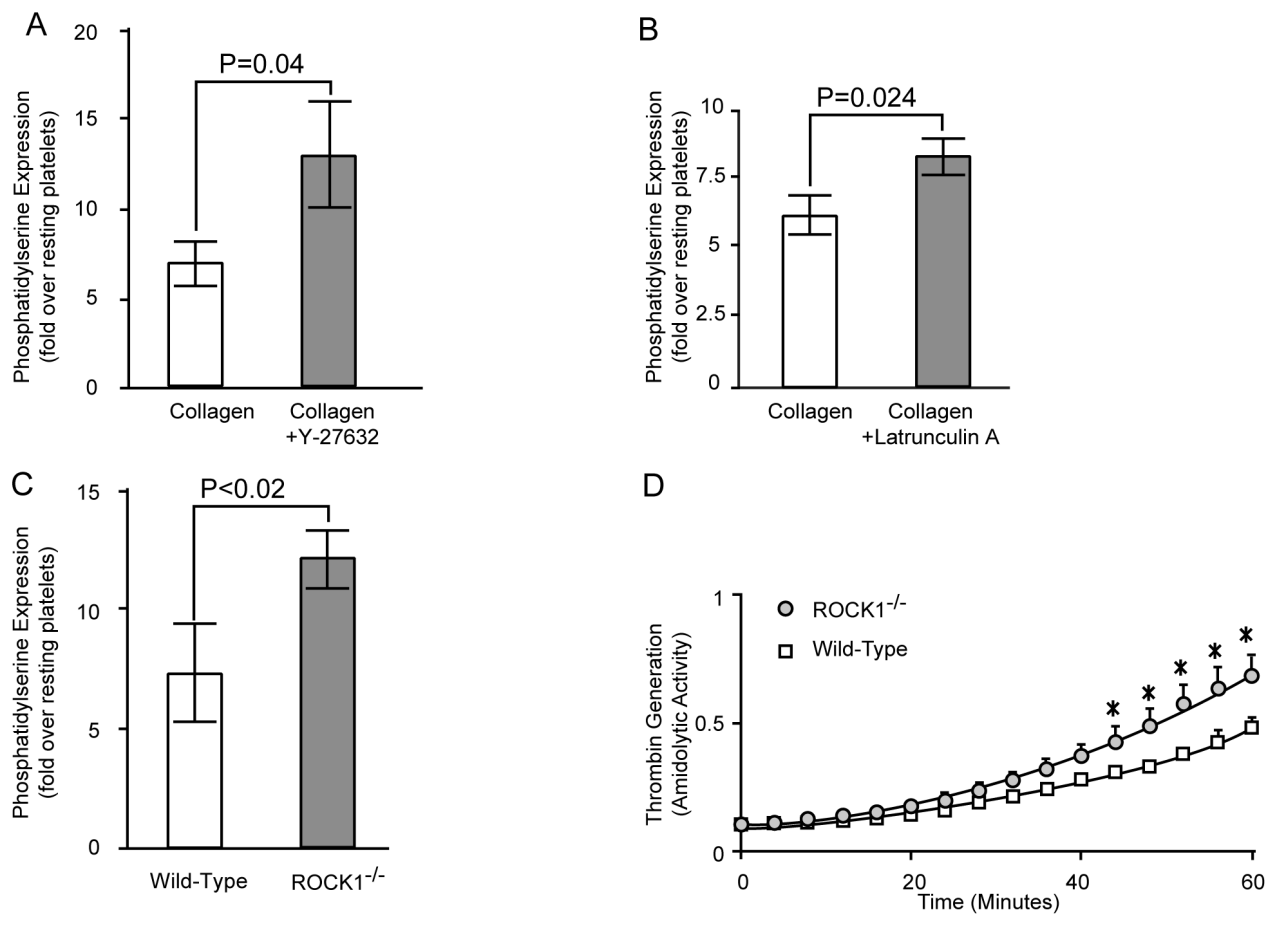
ROCK inhibition and ROCK1-deficiency increases collagen-induced phosphatidylserine expression and thrombin generation. Isolated human (A and B) or murine (C) platelets were stimulated with collagen (10 µg/mL), and FITC-lactadherin and PE-labeled anti-CD42b were added. The exposure of phosphatidylserine was analyzed by flow cytometry. Platelets were incubated with Y-27632 or latrunculin A for 20 minutes before collagen. (D) ROCK1-deficient and wild-type murine platelets were activated with collagen in the presence of prothrombin, factor Xa, calcium, and factor V; thrombin generation was measured by the amiodolyis of thrombin substarte S-2238. (n= 3/group). * denotes a p value of < 0.05.

Consistent with previous studies [8,9], collagen stimulation of human and mouse platelets induced phosphatidylserine exposure (Figure 3). Prior treatment of human platelets with non-specific ROCK inhibitor Y-27632 significantly increased the collagen-induced phosphatidylserine exposure (Figure 3A). Similarly, latrunculin-A, a macrolide toxin that prevents actin polymerization by forming a complex with G-actin [23] also promotes collagen-induced phosphatidylserine exposure (Figure 3B). We then stimulated isolated platelets from ROCK1^-/-^ deficient mice with collagen and found that ROCK1-deficient platelets also exposed significantly more phosphatidylserine when compared to their wild-type controls (Figure 3C). To determine whether the increased phosphatidylserine exposure of collagen-stimulated ROCK1-deficient platelets translated to enhanced procoagulant activity, we measured prothrombinase activity. We found that, when anionic phospholipid was the rate-limiting factor, ROCK1-deficient platelets generated more thrombin compared to wild-type platelets in response to collagen stimulation (Figure 3D). These data indicated that ROCK1 was involved in the increase of collagen-mediated phosphatidylserine exposure and thrombin generation.

**Figure 4:**
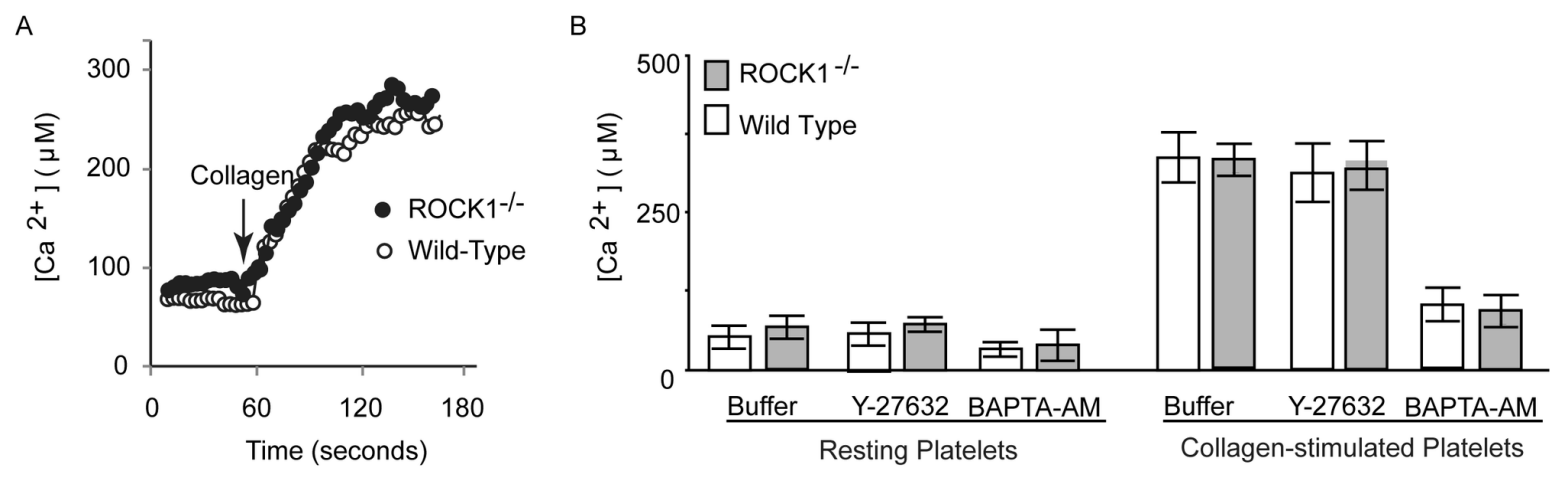
Collagen-induced calcium signaling I s independent of ROCK-1. Fura-2 AM loaded platelets were incubated with buffer, Y-27632 and BAPTA-AM as described in materials and method and activated with collagen. The increase in calcium levels was quantified by measuring Fura-2 fluorescence. (A) Representative traces of Ca^2+^ signaling induced by collagen in isolated murine platelets. (B) Group data of intracellular Ca^2+^ levels in resting and activated platelets (n= 3/group) in presence of buffer, Y-27632 and BAPTA-AM. .

Since the entry of extracellular Ca^2+^ via store-operated calcium channel is an obligatory requirement for phosphatidylserine exposure [24], we measured the increase in intraplatelet Ca^2+^ concentration. As shown in Figure 4A, we found that collagen stimulation significantly increased Ca^2+^ levels in both wild-type and ROCK1-deficient platelets; however, there were no significant differences between the two resting or between the two collagen-stimulated mouse groups. In addition, ROCK inhibitor, Y-27632, had no effect in calcium mobilization (Figure 4B). These data indicated that the increase in phosphatidylserine exposure in response to collagen was downstream of increase in intraplatelet Ca^2+^. 

### ROCK1 is Involved in Platelet F-actin Formation

**Figure 5:**
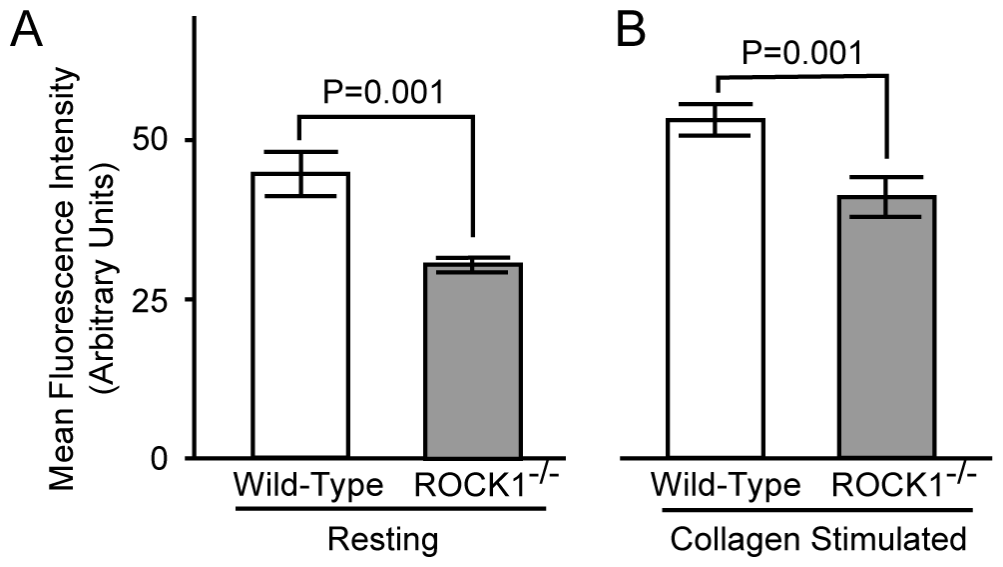
ROCK1-deficiency results in lower F-actin content. Isolated platelets from ROCK1^-/-^ and wild-type mice were stimulated with collagen (10 µg/ml) for 2 minutes; then were fixed, permeabilized, and stained with Alexa Fluor 488-phalloidin, and the F-actin content was quantified by flow cytometry (n= 3/group).

Since ROCK activity was implicated in regulating the polymerization of actin [25], we next assessed F-actin levels in platelets by flow cytometry and found that both ROCK1-deficient resting and collagen-stimulated platelets had decreased F-actin content compared to platelets from wild-type mice (Figure 5). These data indicated that ROCK1 was involved in F-actin formation upon collagen stimulation.

### Lim Kinase-1 and Cofilin-1 are Downstream Targets of ROCK1 in Platelets

**Figure 6:**
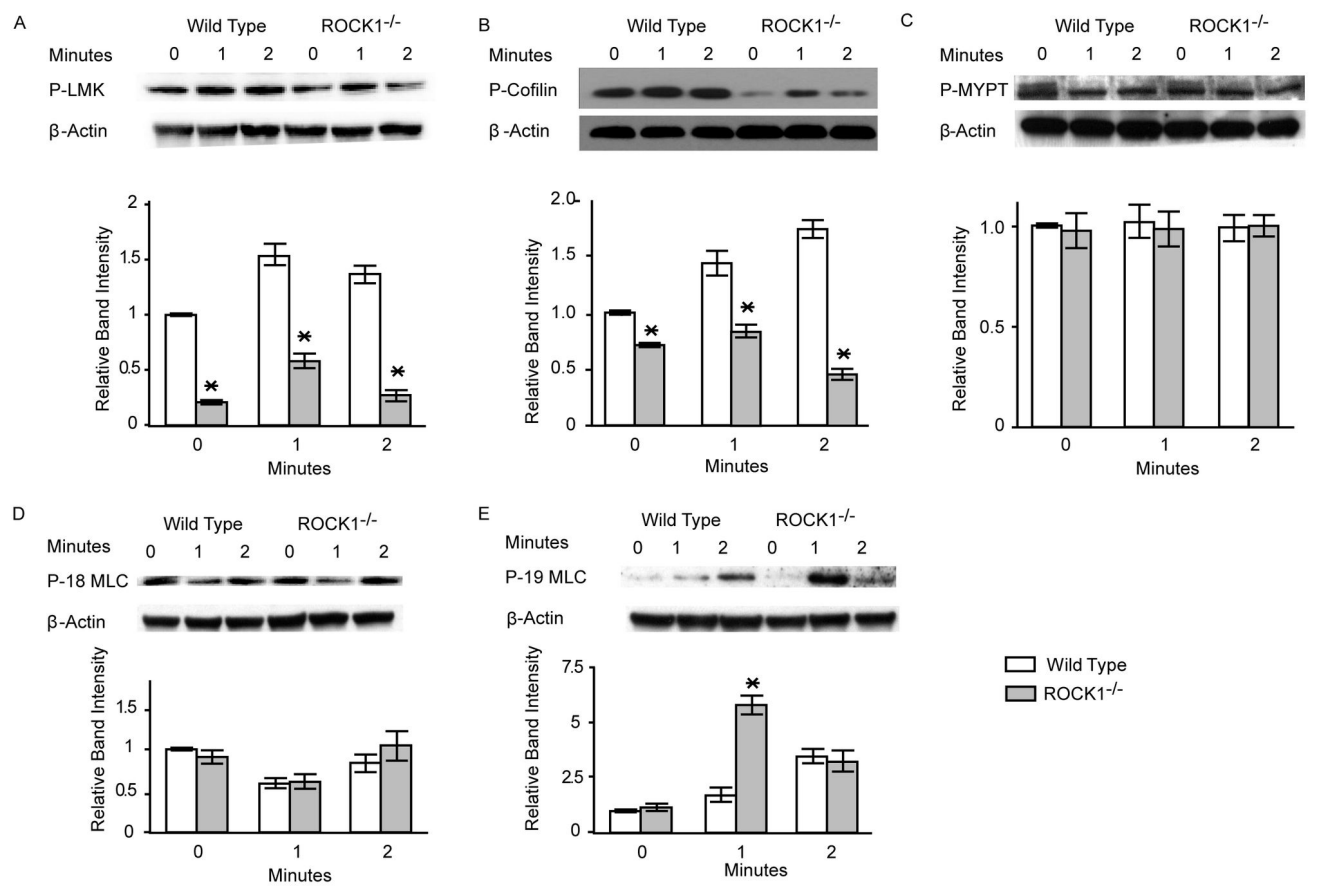
ROCK1-deficieny results in reduced phosphorylation levels. Isolated platelets from ROCK1^−/−^ and wild-type mice were stimulated with collagen (10 µg/ml). Platelets were solubilized at the indicated time intervals, subjected to SDS-PAGE and immunoblotted with antibodies against phospho-Lim Kinase-1 (LMK), phospho-cofilin-1, phospho-myosin phosphatase target subunit-1 (MYPT1); phospho-myosin light chain (threonine 18, P-18 MLC), and phospho-myosin light chain (serine 19; P-19 MLC). Immunoblot of β-actin was used as a loading control. One representative blot and group data (n= 3/group) are shown. * denotes a P value of less than 0.05 compared to wild-type.

To identify possible downstream targets of ROCK1 phosphorylation, we examined the phosphorylation status of several putative ROCK1 substrates in collagen-stimulated ROCK1-deficient and wild-type platelets. As shown in Figure 6A, in ROCK1-deficient platelets we found significantly less phosphorylated Lim Kinase-1 levels than in wild-type platelets. Consistent with this finding, in collagen-stimulated ROCK1-deficient platelets, levels of phosphorylated cofilin-1, a known substrate of Lim Kinase-1 [1], were markedly lower (Figure 6B). Under the same conditions, the phosphorylation levels of MYPT and MLC (threonine 18) were not significantly different between ROCK1-deficient and wild-type platelets (Figure 6C and D). Phosphorylation of MLC (serine 19) (Figure 6E) was even increased in ROCK-1-deficient platelets compared to wild-type cells. These data implicated Lim Kinase-1 and cofilin-1 as possible downstream targets of ROCK1 activity in collagen-stimulated platelets.

### ROCK1^-/-^ Mice are Prothrombotic *in vivo*

**Figure 7:**
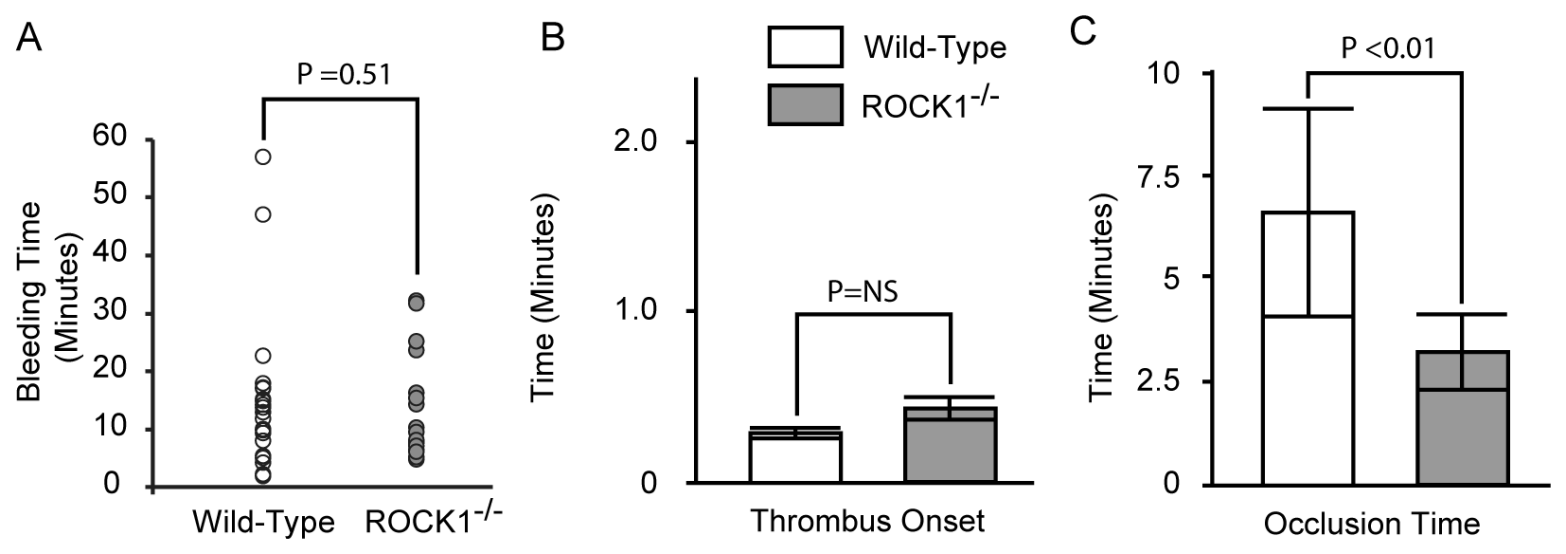
Effect of ROCK1-deficiency on hemostasis *in* *vivo*. (A) Tail-bleeding time. (B and C) Endothelial injury was induced by light/dye in the cremasteric venules and thrombus onset (B) and occlusion time (flow cessation; C) were monitored by intravital microscopy in ROCK1^-/-^ (n= 7) and wild-type mice (n= 8). The P value in (C) was determined by one-way ANOVA.

Since isolated ROCK1-deficient platelets exposed significantly more phosphatidylserine upon collagen activation, we tested the *in vivo* significance of this observation by measuring the tail-bleeding time, as well as the time for thrombus formation and occlusion in cremasteric venules in a light/dye-induced endothelial injury/thrombosis model. We found no significant differences in the tail-bleeding times between ROCK1^-/-^ and wild-type mice (Figure 7A). This is consistent with fact that ROCK1-deficient mice have normal platelet aggregation and secretion and there is no defect in primary hemostasis. Further, we did not observe a significant difference in the initiation time for thrombus formation between ROCK1^-/-^ and wild-type mice (Figure 7B). However, in ROCK1^-/-^ mice the time for occlusion was significantly shorter (Figure 7C). These data indicated that ROCK1 plays a significant role in secondary hemostasis by modulating activation-induced phosphatidylserine exposure. 

## Discussion

Results of this paper show that ROCK1-deficiency accentuated collagen-induced phosphatidylserine exposure in murine platelets. ROCK1-deficient mouse platelets had increased phosphatidylserine exposure and generated more thrombin following activation with collagen. These changes accounted for a shorter occlusion time in a light/dye-induced endothelial injury/thrombosis model and supported the concept that ROCK1 negatively regulated platelet procoagulant responses. It is also possible that changes in the endothelium may play an additional role in the procoagulant response. The most striking effect of ROCK1 deficiency in platelets resulted in the collagen-induced transbilayer movement of phosphatidylserine. There was no significant effect on platelet shape change or aggregation. Several investigators have noticed that the signal transduction pathways for phosphatidylserine exposure were different from those from platelet aggregation and secretion [26]. Patients with impaired secretion have normal phosphatidylserine exposure and platelets from patients with Scott Syndrome, an isolated deficiency of platelet procoagulant activity due to defective phosphatidylserine exposure, have normal aggregation response [27]. 

Collagen interacts with multiple platelet surface receptors including the tyrosine kinase coupled glycoprotein VI and the G protein coupled integrin α2β1to transduce signals via tyrosine phosphorylation of Src and Syk family of tyrosine kinases [28]. Unlike for platelet aggregation, the signaling pathways and the biochemical mechanism of phosphatidylserine translocation across the membrane are poorly understood. Influx of extracellular calcium, via store operated calcium-entry channels [24,29], activates a putative enzyme, scramblase, capable of randomizing anionic phospholipids across the plasma membrane. The precise identity of platelet scramblase is not known. Recently, TMEM16F, a Ca^2+^-activated channel permeable to Ca^2+^ was implicated in Ca^2+^-dependent phospholipid scrambling in blood cells [30,31]. In ROCK1-deficient platelets, there were no significant differences in intraplatelet calcium levels in resting or in collagen-stimulated platelets compared to platelets from wild-type littermates. Furthermore, the ROCK inhibitor, Y-27632, had no effect on collagen induced Ca^2+^ mobilization. Therefore, it is likely that ROCK1 dependant phosphatidylserine exposure is downstream of calcium mobilization. Exposure of phosphatidylserine also occurs in senescent platelets through the mitochondrial Bcl-2 family of proteins [32] and in contrast to collagen induced phosphatidylserine exposure, does not require elevation of cytosolic Ca^2+^ [33] and it involves cytochrome c release and subsequent activation of caspase 3 

Phosphorylation of MLC reflects the contractile activity of actinomyosin. ROCK kinases may increase the phosphorylation of MLC by inhibiting MYPT1 and/or by directly phosphorylating threonine 18 and/or serine 19. In ROCK1 -deficient platelets, there was no significant change in the phosphorylation of threonine 18; phosphorylation of serine 19 was even increased compared to wild-type littermates. The increase in serine 19 phosphorylation may be due to a compensatory increase in MLC kinase activity. ROCK-mediated phosphorylation of MLC was suggested to be involved in platelet shape change [11,12,14]. However, these observations were based on inhibitor studies with Y-27632 and the use of C3-exoenzyme [12] which do not distinguish between the two ROCK isoforms. The normal shape change in ROCK1-deficient platelets suggested that the inhibitory effect of Y-27632 was mediated either by ROCK2 and/or by other Y-27632 sensitive kinases [3,14,16]. Our results indicated that, at least in mice ROCK1 did not mediate platelet shape change in response to collagen. 

The major changes in ROCK1-deficient platelets following collagen stimulation are in the phosphorylation status of cofilin-1. Cofilin-1 plays a pivotal role in actin turnover by depolymerizing existing actin filaments [34]. The actin-binding capacity of cofilin-1 is regulated by phosphorylation at serine-3 and dephosphorylation increases its actin binding [35]. Increased cofilin-1 activity due to dephosphorylation weakens lateral contacts between actin monomers [36] and thereby increases the elasticity of the filaments [37]. In mice, global cofilin-1 deficiency leads to an early embryonic lethality; conditional knock-out of cofilin-1 in megakaryocytes leads to reduced proplatelet formation and production of large platelets displaying an ovoid shape, decreased spreading, and a block in stimulus-induced actin polymerization [38]. 

Actin cytoskeletal assembly plays a major role in maintaining phospholipid asymmetry [39,40]. Many actin binding proteins and cytoskeletal proteins bind to the anionic phospholipid, phosphatidylinositol 4,5-diphosphate (PIP2) and phosphatidylserine which are present exclusively in the inner leaflet [41,42]. Furthermore, F-actin has been shown to bind phosphatidylserine in membranes [43]. These observations suggest that actin fibers are anchored to the cell membrane by their interaction with PIP2 and phosphatidylserine. Focal disruption of this interaction may release constraints on phosphatidylserine allowing its transbilayer movement. In red blood cells, deficiency of the major cytoskeletal protein 4.1, which interacts with phosphatidylserine [44], caused increased phosphatidylserine exposure [45]. During apoptosis membrane fusion and remodeling precedes phosphatidylserine exposure and microvesiculation [40,46]. Thus, in ROCK1-deficient platelets, alterations in the organization of cytoskeleton might release actin fiber-induced constraints on anionic phospholipid on the inner leaflet using similar mechanisms. A possible involvement of actin cytoskeleton in phosphatidylserine exposure was also evident by the observation that the actin depolymerizing agent latrunculin-A promoted collagen-induced phosphatidylserine exposure. 

In conclusion, we have identified a new ROCK1-modulated regulatory mechanism in collagen-induced phosphatidylserine exposure in platelets. This effect is downstream of calcium mobilization and cytoskeletal changes may have mediated this effect 
